# Exploring the experiences and perceptions of haemodialysis patients observing Ramadan fasting: a qualitative study

**DOI:** 10.1186/s12882-021-02255-8

**Published:** 2021-02-02

**Authors:** Nurul Iman Hafizah Adanan, Wan Ahmad Hafiz Wan Md Adnan, Pramod Khosla, Tilakavati Karupaiah, Zulfitri Azuan Mat Daud

**Affiliations:** 1 Department of Dietetics, Faculty of Medicine and Health Sciences, Universiti Putra Malaysia, UPM, 43400 Serdang, Selangor Malaysia; 2 Department of Medicine, Faculty of Medicine, University Malaya Medical Centre, Kuala Lumpur, Malaysia; 3 Department of Nutrition and Food Science, Wayne State University, Detroit, MI USA; 4 School of BioSciences, Taylors’ University, Subang Jaya, Malaysia; 5 Research Center of Excellent Nutrition and Non-communicable Diseases, Faculty of Medicine and Health Sciences, Universiti Putra Malaysia, Serdang, Malaysia

**Keywords:** Ramadan fasting, Haemodialysis, Qualitative, Perception, Experiences

## Abstract

**Background:**

The festival of Ramadan is a month of spiritual reflection for Muslims worldwide. During Ramadan, Muslims are required to refrain from eating and drinking during daylight hours. Although exempted from fasting, many patients undergoing maintenance haemodialysis (HD) opt to participate in this religious practice. Many studies have explored the effects of Ramadan on health outcomes, however, the exploration from patients’ own point of view pertaining to this religious practice is lacking. Thus, we aimed to explore the experiences and perceptions of Muslim HD patients observing Ramadan fasting from three HD centres in Klang Valley, Malaysia.

**Method:**

An exploratory phenomenology qualitative study was conducted whereby subjects were purposively selected based on previous experience in observing Ramadan fasting. Face-to-face in-depth interviews were conducted, and study data were analyzed thematically and iteratively coded using a constant comparison method.

**Results:**

Four major themes emerged from the data, namely: (i) “fasting experiences”, (ii) “perceived side effects of fasting”, (iii) “health-seeking behavior” and, (iv) “education and awareness needs”. Patients expressed the significance of Ramadan fasting as well as the perceived impact of fasting on their health. Additionally, there is lack of health-seeking behaviour observed among patients thus, raising needs for awareness and education related to Ramadan fasting.

**Conclusions:**

Findings of this study shed light on patients’ experiences and perceptions regarding Ramadan fasting which warrants the needs for an effective communication between patients and health care practitioners through a structured-Ramadan specific education program**.**

## Background

Ramadan, the ninth month in the Islamic calendar is celebrated by Muslims globally. During Ramadan, Muslims are required to fast during daylight with smoking, eating and drinking including taking medication as well as engaging in sexual activities being prohibited [1]. However, pre-pubertal children, menstruating, pregnant or lactating women, the elderly, those in poor health, or those in whom fasting may further deteriorate or cause harmful effects to health, are exempted from fasting. As Ramadan is also observed as a month of spiritual reflection, faith and unity, there is high motivation to fasting as an act of religious obligation, even for those who are sick and automatically exempted [2]. In view of the Islamic legal position regarding fasting and HD, there are opposing views of Islamic jurists whether the HD procedure could nullify fasting [3]. According to the Muslims scholars of the Shafi’i school, HD does not invalidate fasting as the procedure is performed via arteries and veins whereby the same legal opinion applies to cupping or *Hijama* [4]. In addition, the insertion of needle at the fistula during HD does not nullify fasting because it is not administered into the body through its open orifices which then reaches the stomach and causes one to feel full [5].

During Ramadan, there is a major shift related to meal timing and meal frequency to accommodate fasting during daylight hours. While fasting hours differs every year depending on the season, Muslims in tropical countries such as Malaysia may experience 13 to 14 h of daylight fasting with average daily temperature of 30 °C every year [6]. These daily lifestyle changes naturally raise concern amongst health care practitioners regarding patients with chronic diseases who still insist on fasting [3]. In particular, end-stage renal disease patients undergoing maintenance haemodialysis (HD) are vulnerable to higher risk for developing malnutrition [7] as well as fluid and electrolyte imbalance [8]. This patient group experiences the retention of uremic waste products due to limited removal via dialysis [9] and a high level of inflammatory cytokines [10] from the catabolic nature of the HD treatment itself. Hence, HD patients have to adhere to prescribed diets with specific fluid and nutrient restrictions [11], that at the same time are adequate for energy and protein to prevent malnutrition [12]. Despite being at risk for malnutrition, around 40 to 70% of HD patients still choose to fast as observed in countries with Muslim populations [13–15]. Reports indicate Ramadan fasting appears to be well-tolerated but careful monitoring of serum electrolyte levels in particular is called for [16, 17] .

Effects of fasting on health outcomes are well researched, but to date no studies have addressed HD patients’ perspectives pertaining to the religious practice of Ramadan. Lack of professional engagement with the patient by the health care practitioner related to cultural beliefs and religious practices may lead to defective health care delivery [18]. The lack of empathy invalidates the concept of a patient-centered care framework as proposed by the Institute of Medicine, whereby physical comfort, emotional support and respectful to patients’ preferences including religious values should be considered to deliver quality health care [19]. The importance of patients’ perspectives and shared roles in health-related decision making rather than passive involvement are highlighted [20] to improve self-efficacy and medical treatment adherence. In fact, patient-centered care has been shown to be positively associated with improved physical and social well-being, as well as increased satisfaction towards health care services [21]. Additionally, achieving spiritual satisfaction and abilities to perform religious duties in individuals generate positive emotions, which in turn increase subjective well-being along with a feeling of respect and acceptance [22]. In this context, failure to explore and understand patients’ preferences leads to poor communication with health care practitioners which can impede patients’ adherence to treatment and dissatisfaction with their medical care [23, 24].

Qualitative studies are advantageous to explore phenomena such as patient beliefs and experiences, which are not usually conveyed in quantitative studies [25]. The experience and perceptions of Ramadan fasting of people with diabetes are well-reported and provide valuable insights [26, 27]. For instance, diabetic patients reported despite experiencing symptoms of hypoglycemia during fasting, they nonetheless continued to fast without consulting their doctors, so as not to feel guilty about not fasting for Ramadan [28]. Some diabetic patients also emphasized that Ramadan fasting was integrated into their lifestyle and it would be sinful not to fast, unless they sensed worsening symptoms during fasting [24, 29]. However, patients with prior knowledge on diabetes management during Ramadan expressed higher confidence and self-reliance in order to make informed decisions regarding fasting [30]. Findings from these studies have been utilized to propose and implement Ramadan education programs enabling diabetic patients to practice safe fasting without compromising their health [31].

With regard to the HD population, quantitative research findings indicate that Ramadan fasting lead to significant changes in nutritional status concerning body weight and biochemical profiles as well as dietary intake [14, 15]. However, the exploration of HD patients’ views on Ramadan fasting practices is unknown. As this issue is a research gap in patient-centered care of the HD population, this qualitative study aims to explore the experiences and perceptions of HD patients observing Ramadan fasting in a Muslim-majority country, Malaysia.

## Methods

### Study design

An exploratory phenomenology qualitative protocol was implemented for this study, whereby in-depth face-to-face interview sessions were conducted with study participants to obtain data. In parallel with the research objective of this study, a phenomenology study aimed to explore and understand a particular life event, in this case Ramadan fasting, from the perspectives of those who had experienced it [32, 33]. In our study, we aimed to recruit HD patients who chose to fast during Ramadan, regardless of the number of fasting days, as our intention was to capture fasting experiences.

### Setting and participant recruitment

This qualitative study involved patients from three HD centres in the Klang Valley, Malaysia who were recruited by criterion purposive sampling. By this sampling method, individuals sharing an experience to a similar phenomenon but who varied as per their individual characteristics and experiences were selected [34]. These were also predetermined characteristics in the selection of sample which was stated in the inclusion criteria of the participants: Muslims patients (*n* = 87) aged between 18 to 60 years old who had participated in the prospective cohort Ramadan-HD Study [15], which evaluated nutritional status during Ramadan from May to June 2018. From this pool, patients with cognitive disabilities and frequent episodes of hemodynamic instability during dialysis such as low blood pressure were excluded from this study to prevent treatment interruption. Potential participants were initially approached during dialysis session, and the study purpose and procedure explained to them, prior to obtaining written informed consent.

### Ethical approval

This study was conducted in accordance with the ethical principles of the Helsinki Declaration, and received ethical approval from the Medical Research and Ethics Committee, Ministry of Health, Malaysia (NMRR-17-2756-37435) and Ethics Committee for Research Involving Human Subjects, Universiti Putra Malaysia. All written informed consent was obtained from subjects prior to the initiation of the study.

### Data collection

**Fig. 1:**
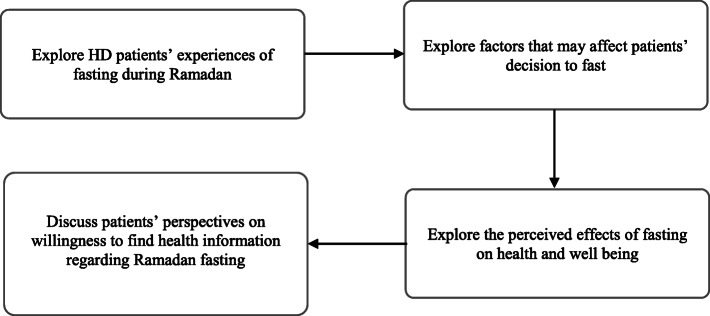
Interview topic framework based on previous literature

Data collection was carried out within 3 months after Ramadan in the year 2019 (July – September 2019) until data saturation point was reached. Data saturation is reached whenever no additional new information can be obtained from study participants [35]. Data collection was conducted during dialysis sessions at respective participating HD units. The interview session was aided by a topic guide which was developed based on relevant literatures regarding Ramadan fasting [26–28, 36]. The interview questions consisted of open-ended questions with prompts. The interview topic framework and flow of sessions is detailed in Fig. 1.

Follow-up questions on participants’ responses were used in order to facilitate and obtain more detailed answers. Two HD patients were selected to pilot the guide in order to refine and finalize the topic guide. The interview sessions were conducted in the Malay language, by a trained researcher with the same cultural and religious background as the study participants to build trust and acceptance to enable a valid, meaningful and in-depth data sharing [37] as well as to prevent misinterpretation of data [38]. This researcher [NIHA] was also trained according to an interview protocol that has been described elsewhere [39]. All interview sessions were conducted by the same researcher. Interviews were audio-recorded and transcribed verbatim along with field notes taken during interview. Data transcripts were verified with the participants’ audio files. Data transcripts were retained in the source language to prevent bias during translation process. In this study, all data transcripts were analysed including additional interviews conducted at the point of presumed saturation in order to verify saturation.

### Data analysis

Final data transcripts were entered into data management software ATLAS.ti 8 (Scientific Software Development GmbH, Berlin) for analysis. This software was used to store, sort and organize data transcripts, as well to annotate and quote phrases in order to generate codes. Transcripts were independently coded by two members of the research team, which also included the interviewer as a member of the coding team. Inductive thematic analysis was used to analyse data transcripts and iteratively coded into themes by using constant comparison method. In this method, the coders initially generated broad ideas (open coding) from each individual data set. Then, a set of central codes were selected through comparison across coding [40]. Codes were matched between independent coders and any discrepancies were discussed until a consensus to derive themes was reached [41]. The initial codes were developed from the individual raw data set by each coder. These codes were then labelled and described by quoting the raw text. At this stage, codes derived by each coder were reviewed until reaching agreement between coders that the code relevant to a theme was appropriate. For example, initially “weight reduction” and “body weakness” were coded separately as positive and negative effects of fasting, respectively. However, the coders agreed that these two codes should be merged under one common theme to ensure all views had the probability on being assigned under relevant themes.

In this study, data collection and analysis were carried out concurrently to achieve inductive thematic saturation, whereby the saturation focused on the emergence of new codes based on the number of existing codes [42]. To determine saturation, we initially identified a set of codes from six interviews as base size (denominator) of the equation [43]. Then, we included two interview sessions for each of the subsequent data runs. The number of new codes emerging from each set of data runs was divided by the denominator. Our data reached saturation when there was < 5% new information threshold at 10th interviews (3.1%) [43]. The first six interviews were selected to produce base codes as ensuing data typically provides the majority of new, high-frequency codes. This has been shown previously with a purposive, homogenous sample that additional subsequent six to twelve interview sessions attained saturation [44].

## Results

### Subjects’ sociodemographic and clinical characteristics

A total of ten (10) interview sessions were required to reach data saturation. The duration of the interview session with subjects ranged between 30 min to 1 h (35 ± 8 min). Gender of the participants was equally distributed (Male/Female = 5/5) and all were from the Malay ethnic group. The mean dialysis vintage for all subjects was 48 ± 22 months with average Kt/V of 1.2 ± 0.2. Although there were noticeably elevated serum potassium and phosphate levels, our participants were generally well during the time of interview. All sociodemographic and clinical characteristics of interviewed participants are presented in Table 1.

**Table 1:** Sociodemographic and clinical characteristics of subjects (*n* = 10)

Sociodemographic background	Mean ± SD Median (IQR)	Frequency (%)
**Gender**		
Male		5 (50)
Female		5 (50)
Age (years)	53 ± 11	
**Marital status**		
Single		1 (10)
Married		9 (90)
**Education level**		
Secondary		6 (60)
Tertiary		4 (40)
**Underlying comorbidities**		
Diabetes mellitus		4 (40)
Hypertension		10 (100)
Hyperlipidemia		3 (30)
Body mass index (kg/m^2^)	27.3 ± 2.7	
**Biochemical indicator**		
Albumin (g/L)	41.4 ± 2.0	
Haemoglobin (g/dL)	11.1 ± 1.2	
Serum potassium (mmol/L)	5.8 ± 1.0	
Serum phosphate (mmol/L)	2.3 ± 0.6	
Dialysis vintage (months)	48 (22)	
Kt/V	1.4 ± 0.2	

### Themes

Four major themes emerged from the data analysis, namely: (1) fasting experiences (2), self-perceived side effects of fasting on general well-being (3), health seeking behavior and (4), education and awareness needs. The subthemes within themes and example of quotes supporting these categories are presented in Table 2.

**Table 2:** Themes, subthemes and supporting quotes

Theme	Subthemes	Supporting quotes
Fasting experiences	• Significance of Ramadan fasting • Factors that affect ability to fast • Support from family members	*“Fasting is an obligation and a norm”* [54 years old, female] *“I’m able to fast but I would skip fasting on dialysis days”* [51 years old, male] *“I usually practice fasting on Monday and Thursday, I’m getting used to it”* [33 years old, female] *“My family members allow me fast, but they did say I can break my fast anytime if I cannot proceed with fasting”* [55 years old, male]
Self-perceived effects of fasting on general well-being	• Positive side effects of fasting • Negative side effects of fasting	*“I feel different. Mainly because I think my weight is reducing if I fast”* [66 years old, male] *“When we fast, we tend to eat little due to early satiety, so I can control my food intake and reduce my body weight”* [38 years old, female] *“The negative part is when you don’t eat, you will feel hungry, uneasiness to the stomach and body becomes weak”* [60 years old, male] *“It’s been a while since I joined congregation Tarawih prayer because I don’t have the energy to do it”* [61 years old, male]
Health-seeking behaviours	• Never sought for health-related advice prior to fasting • Follow previous health advice • Uses internet	*“I feel everything is okay when I fast, so there is no need to ask the staff here”* [54 years old, female] *“In terms of diabetic medication, the doctor did ask me to lower the dosage”*[60 years old, male] *“I had met dietitian when I first started dialysis, I was given a brochure regarding what I can eat so I just followed the advice given until now. It has been a while ago, I cannot remember when”* [66 years old, male]
Education and awareness needs	• Dietary management	*“I wanted to know how much should I eat during suhoor to give enough energy to fast for the whole day”[60 years old, male]* *“What type of food should we take, how much protein, which type food has high phosphate…”*[33 years old, female]

Findings related to the themes are described below, with subjects identified only by age and gender, with an asterisk (*) to indicate the same subject had been quoted more than once,

#### Theme 1: fasting experiences

There were three subthemes identified within the theme ‘fasting experiences’. Firstly, participants described the significance of fasting to them. They also identified factors that could affect the ability to observe fasting on a daily basis. Subjects also discussed on the role of family support in the decision making to fast during Ramadan.

##### Subtheme- significance of Ramadan fasting

All interviewed participants emphasized the significance of Ramadan fasting as a religious obligation that should be fulfilled despite having to undergo HD treatment. About one third of participants also viewed that Ramadan fasting is a religious norm for all Muslims that is practiced every year. Despite having a history of being critically ill in the past, one participant also described that she would still fast for the coming years as her health condition had improved.

*“Fasting during Ramadan is an obligation and a norm for Muslims.”* (60 years old, male)*

##### Subtheme- factors that affect ability to fast

Participants were equally divided on those who were able to fast for one whole month and those who did not. More than half of the participants stated that the ability to fast was determined by their health condition on a daily basis during Ramadan. Another participant also stated that although he is able to fast, he would usually skip fasting on dialysis days due to fear of the side effects of fasting. He also reported that he had consulted the religious official that it would be permissible to skip fasting if a dialysis patient, with the condition that a sum of money or food known as *fidyah* would be given to those in need, for each fasting day missed. A female participant stated that although she observed fasting during Ramadan she would break the fast should there be any health concerns arising such as extreme fatigue or hypoglycemia. These findings indicate that majority of our participants were able to recognize both religious obligations as well as health-related concerns pertaining to fasting.

*“I am able to fast, but I skip fasting on dialysis days. I did try to fast while doing dialysis, but I would feel very exhausted. ”* [51 years old, male]*

##### Subtheme- family support

In terms of family support, four participants stated that there was no pressure from family members to perform fasting. According to them, family members also showed support in decision-making as long as their health condition permitted, as indicated by this viewpoint:

*“There was no pressure from family members because it all depends on our own health condition”* [60 years old, male]*

There was one participant who claimed that her family was once against her fasting years ago due to her poor health condition but as her condition improved, the family then supported her to fast. This was reflected in her statement:

*“It was back in 2016 or 2017 that I did not manage to fast because my health condition was bad, my children were against it too. But in the most recent years, as my health condition is getting better, I managed to fast. I was even able to complete one month of fast”* [65 years old, female]

There were also participants who stated that their family members supported their decision regardless of whether they decided to observe fasting or not. These participants stated that they also received neither pressure nor judgement from family members regarding their decision to fast.

#### Theme 2: self-perceived side effects of fasting on general well-being

All participants perceived that fasting impacted their general well-being. Under this theme, two subthemes indicated the positive and negative perceived effects of fasting.

##### Subtheme- positive side effects of fasting

Generally, majority of the participants found that Ramadan fasting lead to reduction of body weight. This was mainly related to reduction of food and fluid intake during daylight in Ramadan. These participants claimed feeling “lighter” as a result of fasting which then gave them a positive feeling, as indicated by this comment:

*“I feel healthier when I fast. My body also feels lighter”* [51 years old, male]

Another participant also mentioned that fasting could help to suppress overall appetite and lead to further ability to control food intake during the breaking of fast, which then further led to reduction of body weight during Ramadan. This statement was:

*“I don’t usually eat much at the breaking of fast due to early satiety. I feel like I am able to control my food intake, thus it is how I lose weight”* [33 years old, female]*

In addition, some of the participants also claimed that fasting lead them to have better fluid control compared to non-fasting days, as one comment indicated:

*“The positive thing about fasting is my daily weight increment is only about 200 to 300 g as compared to my dry weight”* [55 years old, male]

##### Subtheme- negative side effects of fasting

More than half of the subjects [7/10] agreed that abstinence from eating and drinking lead to feeling tired and body weakness and the symptoms usually peaked at mid-day but resolved after the breaking of fast. This was a typical comment:

*“Only that when we are fasting, we feel hungry and give discomfort to the stomach and body becomes weaker”* [60 years old, male]

On the other hand, one subject with underlying diabetes additionally claimed that he would break his fast if there was presence of any symptoms of hypoglycemia as advised by the doctor. His comment was:

*“If I had hypo while fasting, I would break my fast as advised by the doctor. But this happened to me a while ago”* [55 years old, male]

#### Theme 3: health-seeking behavior

This theme concerned whether participants ever sought for health-related advice or information prior to Ramadan. Three subthemes identified were (1): never sought any health-related information regarding fasting (2), follow previous health advice from health care professional and (3) seeking information through internet.

##### Subtheme- never sought health-related information

Almost all participants [8/10] claimed they did not seek any health-related information or advice regarding fasting as it had become a routine and they did not face any health problem as a result of fasting, as indicated by this comment:

*“I feel well when I fast, and I do not experience any problem while fasting. So, I don’t think there is a need for me to ask advice from the staff or doctors about this matter”* [54 years old, female]

##### Subtheme- follow previous health advice

One participant with diabetes claimed that he had been advised on insulin adjustment by the doctor in the past years. Accordingly, he just followed the advice whenever he was planning to fast, whether on Ramadan or regular days, as given by this statement:

*“In terms of medication, the doctor has previously advised me to lower the dosage and not to inject insulin while I am fasting”[60 years old, male]*

Only two of the participants reported that they had seen a dietitian at least once after initiating dialysis treatment whether at the hospital or dialysis centers. However, when further probed if they consulted pertaining to Ramadan, none of them declared they consulted regarding dietary management, as reflected by this viewpoint:

*“I had seen dietitian few years ago when I first started my dialysis, so I just refer the dietary advice given”* [66 years old, male]

##### Subtheme- seeking information through internet

Two participants reported that they had utilized the internet to seek health and Ramadan related information. However, these participants also stated that there were no specific website with information regarding Ramadan fasting for HD patients, as reflected by this statement:

*“Usually I just Googled whenever I want to find information. For example, I just typed in haemodialysis and fasting” [38 years old, female]*

#### Theme 4: education and awareness needs

In general, all participants agreed that health-related education and awareness should be emphasized in order for them to practice safe fasting. Although none of them had experienced severe side effect of fasting, they claimed that education and awareness should be given to patients especially in terms of dietary management in order to prevent health-related complications. Thus, they suggested that a more specific education and awareness regarding Ramadan fasting should be given to HD patients.

##### Subtheme- dietary management

Most participants [7/10] reported they did not know the right amount of food to be taken at *suhoor,* which is the meal consumed at dawn to ensure sustenance of energy for fasting especially on dialysis days. They felt that this issue should be addressed through providing a dietary guideline for *suhoor*, before fasting commences, if they decided to observe fasting on dialysis days. The patient consensus was revealed by this typical comment:

*“I really wanted to know how much I should eat at suhoor because I did not know whether I am eating enough to sustain energy”* [60 years old, male]

Many participants [6/10] also stated that there was lack of information on the quality and quantity of food to be consumed at the breaking of fast for dialysis patients. As Ramadan is usually celebrated as a festival where there will be a variety of foods being served in a buffet and at bazaars, these participants said they needed more guidance on how much they should eat. Their family members would also bring take-away foods home, which sometimes was hard to resist. This statement reflected their views:

*“Information like how much of protein should I take, which type of food has high phosphate. Those are the things I wanted to know” [33 years old, female]*

## Discussion

In this study, participants’ experience during Ramadan with regard to their decision to fast were influenced mainly by religious obligation, support from family members as well as their health status. This view of Muslim HD patients resonates with a previous qualitative study with diabetic patients which revealed Ramadan fasting was viewed as a religious duty to be carried out by Muslims despite having diabetes [28]. It appears that this study’s HD patients also concurred with this view on religious duty, and felt confident to observe fasting as a norm to fulfil spiritual needs in their life. However for those with chronic diseases, Ramadan fasting lasting one month may present obstacles and hardships as the abstinence from eating and drinking for more than 10 h requires restrain and discipline [36]. In our study, most participants claimed that their family gave moral support regarding their decision to fast, depending on their health status. Social support particularly from family members seems important to assist patients to fulfil religious duty and managing their disease [45]. In addition, being able to perform religious duty with the support of others can provide people with a sense of achievement, thus giving positive impact to overall well-being [46].

In terms of self-perceived effects on general well-being, our study participants considered fasting as beneficial to their mental health and physical well-being. They recognized the most beneficial effects towards physical health were fluid and body weight control, believing that dietary control mediated by eating less during Ramadan compared to regular days played an important part in this overall well-being. Interestingly, diabetes patients also reported that Ramadan fasting contributed to a better glucose management with the perception that a better dietary control was afforded through limited meal frequency during Ramadan [47]. This finding is consistent with quantitative studies on the HD population showing body weight and interdialytic weight gain (IDWG) decreased significantly during Ramadan for fasting patients [15, 48]. IDWG control is critical to HD patients if greater than 4% of total dry weight which may induce higher ultrafiltration rate subsequently leading to increased risk of cardiac mortality for these patients [49]. However, reduction of total body mass index (BMI) should be misinterpreted as a false positive effect because low BMI could be indicative of muscle wasting associated with higher mortality risk for this patient group [50].

Our study indicates that issues pertaining to dietary intake confers an important area of concern to the fasting patients. Although majority of our participants reported that they had better control of food intake due to limited meal timing during fasting, it is important to note that quantitative analysis indicates HD patients did not achieve their required dietary recommendations during Ramadan especially protein intake [47], and had a tendency towards practicing dietary monotony [51]. Long-term dietary inadequacy may predispose HD patients to increased risk of developing protein-energy wasting [52]. Therefore, appropriate dietary advice should be given by dietitians to emphasize on adequacy of energy and protein intake whilst moderating specific nutrient restrictions such as phosphorous and potassium during Ramadan. On the other hand, the most commonly reported side effects of fasting by our study participants were body weakness and lethargy due to prolonged abstinence from eating and drinking. In fact, one participant was unable to fast on dialysis days due to inability to cope with fatigue following HD treatment. These symptoms such as tiredness, feeling sick and dehydrated have been commonly reported across other Muslim populations observing Ramadan fasting [27, 28, 47].

Of great concern, we found that majority of the participants never sought any advice regarding fasting from healthcare professionals. Their confidence was based on no major detrimental health issues occurring during previous Ramadan fasting. This finding is also consistent with observations on different populations whereby patients did not seek health advice prior to fasting, and self-adjusted their medication regime without consulting their doctors [24, 28]. In fact, they only relied on their past experiences of fasting success [53]. In addition, many patients also chose not to inform their health care practitioners before commencing fasting in the fear of been advised against fasting and lack of understanding from doctors [28, 54]. Some patients in this study also claimed that they had learnt to manage diets during Ramadan fasting and deal with issues of hypoglycemia over time.

This study highlights some important new findings. Firstly, the strong desire to participate in Ramadan fasting may raise needs for awareness among healthcare practitioners to ensure these patients can practice fasting safely [24]. Furthermore, this study also found that most of the patients did not consult their doctors before commencing fasting. Ideally, patients should be informed about the importance of consulting their clinicians before Ramadan to enable fasting safely, especially for those with long term chronic diseases. Lack of access to healthcare providers whom patients can easily trust and communicate with is a barrier to self-efficacy [55]. Thus, healthcare practitioners should adopt various strategies in order to promote patients’ self-efficacy such as through self-management programs or training [56] tailored to patients’ individual needs or allow them to make their own decision based on their belief practices [57]. Besides, other non-traditional strategies via telehealth, mobile application or the social media may also be utilised as a platform to share resources and provide support to patients with chronic diseases [55]. The implementation of these strategies will not only improve communication between patients and their healthcare practitioners regarding Ramadan fasting, but also improve patients’ self-efficacy towards managing their disease.

Secondly, as dietary intake plays an important role during Ramadan and should be managed according to restrictive dietary requirements to maintain metabolic control for the HD population, our participants emphasized that a more specific education and information regarding dietary management specifically tailored for Ramadan should be available to them. This includes the quality, quantity and type of foods permitted during this fasting month. Other quantitative studies have indicated that although total dietary energy and protein intakes decreased significantly during Ramadan, there was a notably increased intake of dietary potassium attributed to food and beverages with high potassium [14, 15] content such as dates and fruit juices which are typically consumed during the breaking of fast [58].

A Ramadan-focused education program should serve as bridge to the gap in health and nutrition-related knowledge, as well as addressing patients’ religious responsibilities and spiritual needs [59]. For instance, a previous study using a structured education given prior to fasting, found it improved the quality of fasting among type 1 diabetes patients, in the sense of improvement in self-reliance as well as reduction of adverse events during fasting [30]. This provides evidence showing education programs empower patients to make informed decision and improve disease management in relation to the knowledge and skills attained through the program [60]. To date, no guidelines have been published specifically for kidney patients related to Ramadan fasting. Thus, comprehensive practical guidelines that provide detailed risk stratification for patients, as well as pre- and during Ramadan education that includes both nutrition and medical advice will further empower healthcare practitioners to deliver the best care and support to patients [31]. In order to plan for a Ramadan-focused education or practical guideline, clinical and cultural competencies of health care practitioners [60] are needed in order to counsel patients appropriate to health and spiritual needs [27]. The lack of communication in relating to Islam and Ramadan fasting practices among healthcare practitioners may also hinder effective care delivery to patients [61]. In Malaysia, there is an absence of formalised opinion to guide healthcare practitioners on handling Ramadan fasting intentions of patients. Therefore, medical practitioners tackle this issue on an individual basis, depending on the literatures to inform on decisions. As such, a specific communication tool such as RAMCOM should be able to assist healthcare practitioners in order to communicate with Muslim patients wishing to observe Ramadan fasting [62]. The RAMCOM communication tool also allows for shared decision making related to fasting as well as addressing common issues that may arise such as medication dosage adjustment, signs and symptoms arising during fasting, as well as lifestyle changes during Ramadan. A patient-centered education program in parallel with effective communication between patients and health care practitioners, could increase health self-efficacy and adherence to medical treatment leading to improved health outcomes [63, 64].

To the best of our knowledge, this is the first qualitative study to explore experience and perceptions of maintenance HD patients during Ramadan fasting. We also identified a range of topic guides of patients’ experiences and perceptions including their views on fasting, support from family members, as well as their health seeking behaviours which have not been explored before. This study also revealed the needs for awareness and a structured Ramadan-focused education program tailored for the HD population to ensure that fasting can be practiced in the safest way possible enabled by their health care practitioners.

However, our study has several limitations. Firstly, this study was conducted with the Malay ethnic group which are largely residing in Malaysia, Brunei, Singapore and Indonesia. Although data from ten participants were sufficient to reach data saturation in this population, these views may not be generalized to a broader Muslim HD population such as in the Middle Eastern Region and in Muslim-minority countries. Besides, this study included patients who have been on HD treatment for a long period. Patients who have just initiated their HD treatment may have a priority to adjust to metabolic shifts in clinical disease management that prevails when transitioning from Stage 4 to Stage 5 CKD, and face high risk for mortality from poor nutritional status [65, 66]. It is also beyond the scope of the current study to further explore the influence of socioeconomic status, education background as well as family structures on patients’ awareness regarding religious practices such as fasting and receptivity towards medical advice. Patients with higher socioeconomic status may have higher self-awareness and better access to health care which eventually influences their satisfaction towards quality health care [67]. Additionally, as limitations are inherent to the phenomenological approach, this study only included patients with relatively higher than average educational levels and those who chose to fast during Ramadan. A more comprehensive views pertaining to Ramadan fasting would have been generated if patients with lower education status and who did not fast were included. This study also excluded individuals undergoing peritoneal dialysis as these patients do conventionally observe Ramadan fasting. Lastly, this retrospective study that is based on interviews also may be prone to memory bias compared to actual fasting experience.

## Conclusions

As a conclusion, this study has explored four main findings regarding HD patients’ experiences and perceptions on Ramadan fasting. The findings of this study should be able to bridge the current research gap in terms of exploring patients’ views pertaining to Ramadan fasting; thus enabling improved patient care and building effective communication with health care practitioners. The findings of this study should assist health care practitioners in communicating with patients regarding their decision to fast. As there are large numbers of Muslim HD patients observing fasting, it is critical for health care practitioners to incorporate both clinical and cultural competencies in their practice so as to build trust and confidence with their patients who seek information regarding safe fasting. In addition, a Ramadan-focused patient-centered education awareness program should be implemented to improve patients’ knowledge and disease management skills to ensure safe fasting. This awareness program can be developed through various authorities including health care professionals and religious authorities as suggested in a previous study. Consequently, this Ramadan-focused education program should be evaluated to identify benefits and effectiveness.

## Data Availability

The datasets generated and/or analysed during the current study are available from the corresponding author on reasonable request.
